# Climate acts as an environmental filter to plant pathogens

**DOI:** 10.1093/ismejo/wrae010

**Published:** 2024-01-23

**Authors:** Maria Caballol, Miguel Ángel Redondo, Núria Catalán, Tamara Corcobado, Thomas Jung, Benoît Marçais, Ivan Milenković, Miguel Nemesio-Gorriz, Jan Stenlid, Jonàs Oliva

**Affiliations:** Department of Agricultural and Forest Sciences and Engineering, University of Lleida, Lleida 25198, Spain; Joint Research Unit CTFC – AGROTECNIO-CERCA, Lleida 25198, Spain; Department of Forest Mycology and Plant Pathology, Swedish University of Agricultural Sciences, PO Box 7026, Uppsala 750 07, Sweden; Institute of Environmental Assessment and Water Research, IDAEA-CSIC, Barcelona 08034, Spain; Mendel University in Brno, Faculty of Forestry and Wood Technology, Department of Forest Protection and Wildlife Management, Phytophthora Research Centre, Brno 613 00, Czech Republic; Austrian Research Centre for Forests (BFW), Seckendorff-Gudent-Weg 8, Vienna 1131, Austria; Mendel University in Brno, Faculty of Forestry and Wood Technology, Department of Forest Protection and Wildlife Management, Phytophthora Research Centre, Brno 613 00, Czech Republic; Université de Lorraine - INRAE, UMR Interactions Arbres/Microorganismes, Nancy 54000, France; Mendel University in Brno, Faculty of Forestry and Wood Technology, Department of Forest Protection and Wildlife Management, Phytophthora Research Centre, Brno 613 00, Czech Republic; University of Belgrade-Faculty of Forestry, Belgrade 11030, Serbia; Forestry Development Department, Teagasc, Dublin D15DY05, Ireland; Department of Forest Mycology and Plant Pathology, Swedish University of Agricultural Sciences, PO Box 7026, Uppsala 750 07, Sweden; Department of Agricultural and Forest Sciences and Engineering, University of Lleida, Lleida 25198, Spain; Joint Research Unit CTFC – AGROTECNIO-CERCA, Lleida 25198, Spain

**Keywords:** climate change, competitive exclusion, drought, forest pathogen, functional diversity, functional traits, oomycete, Phytophthora, species distribution model, temperature

## Abstract

Climate shapes the distribution of plant-associated microbes such as mycorrhizal and endophytic fungi. However, the role of climate in plant pathogen community assembly is less understood. Here, we explored the role of climate in the assembly of *Phytophthora* communities at >250 sites along a latitudinal gradient from Spain to northern Sweden and an altitudinal gradient from the Spanish Pyrenees to lowland areas. Communities were detected by ITS sequencing of river filtrates. Mediation analysis supported the role of climate in the biogeography of *Phytophthora* and ruled out other environmental factors such as geography or tree diversity. Comparisons of functional and species diversity showed that environmental filtering dominated over competitive exclusion in Europe. Temperature and precipitation acted as environmental filters at different extremes of the gradients. In northern regions, winter temperatures acted as an environmental filter on *Phytophthora* community assembly, selecting species adapted to survive low minimum temperatures. In southern latitudes, a hot dry climate was the main environmental filter, resulting in communities dominated by drought-tolerant *Phytophthora* species with thick oospore walls, a high optimum temperature for growth, and a high maximum temperature limit for growth. By taking a community ecology approach, we show that the establishment of *Phytophthora* plant pathogens in Europe is mainly restricted by cold temperatures.

## Introduction

Climate change and forest pathogens pose a combined and often synergistic threat to natural ecosystems worldwide [1, 2]. Climate has been suggested to explain the composition of microbial communities such as bacteria [3], mycorrhizal fungi [4–6], and plant endophytes [7]. Many studies have predicted an expansion of plant pathogens with climate change based on single-species models [8, 9]. However, predictions based on models considering pathogens as independent and static entities may not fully represent the complexity of their interactions in nature. Plants are usually affected by more than one pathogen at the same time and pathogens may infect more than one host plant at a time. In addition, plant pathogens compete with native and non-native species, which may or may not have reached their potential niches yet. Furthermore, both host distribution and invasion do not happen stochastically and may also follow their own climatic signal.

The potential effect of climate change on plant pathogens can be assessed from a community ecology perspective. In general, plant pathogens are distributed across different phyla across fungal and oomycete kingdoms, which complicates comparisons in terms of niche or function. However, this is not the case for the oomycete genus *Phytophthora*. The genus *Phytophthora* comprises an ecologically diverse array of species (ca. 200 species in total). Some of these species are responsible for global plant disease outbreaks [10–12], some have wide-host ranges whereas others are more specific, and some have low pathogenic potential [12–14]. Furthermore, the genus *Phytophthora* provides some of the clearest examples of the introduction and spread of invasive pathogens via the trade of living plants and germplasm and other anthropogenic activities [15–18]. Last but not least, the number of *Phytophthora* species inhabiting a certain area can be estimated based on their presence in watercourses downstream, thus bypassing the challenge of detecting these cryptic organisms directly from plant tissues and soil samples [19].

Mechanisms underlying pathogen responses to climate can be predicted from their functional traits [20–22]. Nevertheless, the use of functional traits in pathogen ecology remains testimonial. For example, the inability of certain species to produce survival structures has been regarded as a major impediment to their surviving cold conditions [20, 21]. For analogous reasons and because *Phytophthora* species require free water for spread and infection via swimming zoospores [13, 23–25], traits that improve the capacity of *Phytophthora* species to cope with dry conditions have also been regarded as crucial for their survival [26, 27]. However, whether the distribution of *Phytophthora* can be predicted from functional traits at the community level or if those traits are related to environmental stress is largely unknown. Recently, *Phytophthora* species were annotated with ca. 30 functional traits [28, 29], including traits related to environmental stress and others that reflect differences in terms of specialization or residence time (i.e., the number of years since they were first reported in a certain area).

Understanding whether climate represents a significant environmental filter for pathogen communities could also improve our predictions regarding the effect of climate change on pathogen distribution. Testing whether the functional richness of a pathogen community is lower or greater than expected given the observed species richness should theoretically differentiate environmental filtering from competitive exclusion [30]. The rationale for this principle is that under environmental constraints, certain functional traits would be selected and species would tend to be more similar to each other [30–32]. By contrast, unconstrained conditions would facilitate competition and trait diversification amongst species [30, 31]. One study in Northern Europe showed that community assembly depended on functional traits linked to climatic requirements and to the ability to form survival structures [20]. Although surviving harsh conditions seems to be a key trait for species to colonize northern latitudes [8, 20, 21], it is not known which mechanisms may predict community assembly in southern latitudes.

In this study, we investigated the role of climate on the assembly of plant pathogen communities of the genus *Phytophthora* at a geographical scale and across bioclimatic regions and included Mediterranean, continental, oceanic, subalpine, boreal, and Arctic conditions. We tested whether the biogeography and seasonal variation of *Phytophthora* was linked to climatic variables and whether other environmental factors played a role. We also investigated whether the influence of climate on species differed depending on whether they were introduced a long time ago or introduced recently. We also explored whether and where environmental filtering or competitive exclusion occurred in Europe. Finally, we investigated whether distribution could be predicted from functional traits.

To study these questions, *Phytophthora* communities were determined by sequencing the internal transcribed spacer (ITS) region of water filtrates from forest rivers and streams. Samples were obtained from 118 streams along an altitudinal gradient in Catalonia (NE Spain) from the Pyrenees mountains to lowland areas close to the sea, and from 183 stream sites along a latitudinal gradient from Spain to northern Sweden in a similar type of catchment. The influence of seasonal variation on the altitudinal gradient samples was assessed by re-sampling the 118 streams for two consecutive years in spring and autumn. Diversity and functional diversity of *Phytophthora* communities were correlated with climatic variables and alternative explanatory variables such as tree diversity and water chemistry. Species were classified as introduced a long time ago or recently introduced based on the date on which they were first reported, which was used as a proxy for residence time. Signs of environmental filtering and competitive exclusion were obtained by performing randomization analysis.

## Material and methods

### Stream surveys

**Figure 1:**
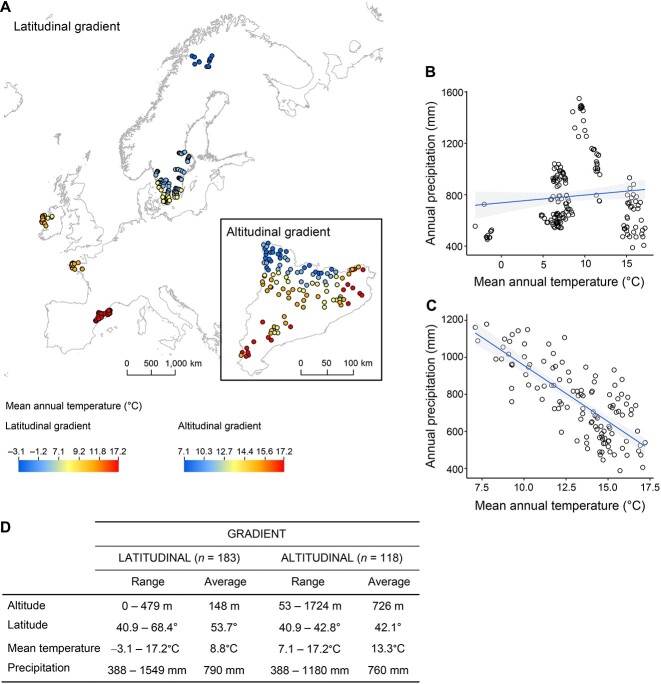
**Stream site locations and geographic and climate characteristics along a latitudinal gradient and an altitudinal gradient.** (A) Location of streams surveyed along the latitudinal and altitudinal gradients. (B, C) Correlation between annual precipitation and mean annual temperature along (B) the latitudinal and (C) the altitudinal gradient. (D) Geographic and climatic variables along the latitudinal and altitudinal gradients.

We surveyed a total of 263 streams along two gradients, i.e., altitudinal and latitudinal (Fig. 1A). For the altitudinal gradient survey, 118 streams from independent watersheds were sampled in Catalonia (NE Spain) in the spring and autumn in 2018 and 2019 (Fig. 1A, Supplementary Information Fig. S1). The latitudinal gradient survey comprised 183 stream sites ranging from NE Spain to northern Sweden (Fig. 1A). Both the altitudinal and latitudinal gradients covered a range of climatic conditions (Fig. 1D). Although no correlation between temperature and precipitation was found along the latitudinal gradient (Fig. 1B), temperature and precipitation were negatively correlated along the altitudinal gradient (Fig. 1C).

Sampling was carried out by collecting 6 l of water at each site, which were subsequently filtered through an 8-μm membrane (Merck Millipore, Cork, Ireland) attached to the pump of an agricultural hand sprayer with a polysulfone filter holder [20]. During the altitudinal gradient survey in the spring of 2019, water samples were collected at each sampling site to determine water chemistry parameters. A detailed explanation of the stream surveys and water analysis can be found in the Supplementary Information Methods S1.

### Library preparation for ITS sequencing

For DNA extraction, filters were cut in half and DNA was extracted using a NucleoSpin Soil kit (Macherey-Nagel, Düren, Germany). For filters from samples collected in France, DNA was extracted using Lysing Matrix A tubes (MP Biomedicals, Illkirch, France) and a DNeasy extraction kit (Qiagen, Hilden, Germany). Libraries were prepared using tagged primers [20], based on *Phytophthora*-specific primers [33] as previously described [34]. Seven pools comprising the 512 samples collected in Spain, France, Ireland, and northern Sweden were sequenced. Each of these pools was sequenced in a SMRT PacBio cell at SciLifeLab (Uppsala, Sweden). Samples collected in southern Sweden were prepared by Redondo et al. [20] and sequenced in 16 SMRT PacBio cells at SciLife Lab (Uppsala, Sweden). In total, 704 samples were sequenced using 23 SMRT cells, yielding a total output of 2 220 242 reads.

### Bioinformatic analysis of sequencing data

Sequences were de-multiplexed, filtered, and clustered using the SCATA pipeline (http://scata.mykopat.slu.se). Quality control was performed to keep only reads with an average score of >10 or with a base quality of >2, containing both primers (using 0.9 as a primer match) and longer than 690 bp and smaller than 820 bp. A total of 785 385 reads passed quality control. After that, reads were clustered in operational taxonomic units (OTUs) using a clustering distance of 0.001 and a minimum length for pairwise alignment of 0.85. Samples with different tags in the forward and reverse primers were deleted. OTUs were identified by performing a BLAST analysis of the consensus sequence of each cluster with the ITS reference sequences for *Phytophthora* spp. from *Phytophthora*-ID (http://Phytophthora-id.org) and the ITS reference sequences for *Halophytophthora* and *Nothophytophthora* spp. from GenBank (http://www.ncbi.nlm.nih.gov/genbank/). A minimum threshold of 99.8% identity and 90% coverage was established. When an OTU was assigned as both *Phytophthora plurivora* and *Phytophthora citricola*, we used MEGA software (version 11) [35] to align the consensus sequence of the OTU to the ITS sequences of *P. plurivora* and *P. citricola* type isolates from Jung and Burgess [36] to assign the OTU to one of these two closely related species.

### Climatic and vegetation data

Monthly climatic data for the altitudinal gradient sampling plots were obtained from the Meteorological Service of Catalonia website (https://www.meteo.cat) for 1976 to 2020. Monthly climatic data for the latitudinal gradient sampling plots (i.e., plots in France, Ireland, and Sweden) were obtained from the KNMI Climate Explorer webpage (https://climexp.knmi.nl/start.cgi) for 1975 to 2021. We computed the average seasonal temperature and precipitation over the available period as well as the average annual temperature and total annual precipitation as described in the Supplementary Information Methods S2.

For each stream of the altitudinal gradient survey, tree diversity was computed with the Shannon index for the riverbank vegetation and the Shannon index for the watershed vegetation. The presence (%) of the most dominant tree genera in the watershed and on the riverbank were also used as plant variables. Moreover, we calculated the following topographical parameters: the proportion of each type of land cover (i.e., urban, agricultural, or forest), the total watershed area, aspect, and slope. The followed procedures are described in the Supplementary Information Methods S2.

### Distribution of *Phytophthora, Halophytophthora,* and *Nothophytophthora* species

We analysed the association between the relative abundance of the different *Phytophthora, Halophytophthora,* and *Nothophytophthora* species (proportion of reads belonging to a species in the sample) in the altitudinal and latitudinal gradient plots and climate. Analyses of the autumn and spring samples from the altitudinal gradient plots were conducted separately because the relative abundance and presence of many species differed between the two sampling seasons. For the altitudinal gradient survey, we also analysed the association between the relative abundance of *Phytophthora, Halophytophthora,* and *Nothophytophthora* species and other environmental factors, such as topographical parameters, tree diversity, and water chemistry parameters. The relationship between the relative abundance of *Phytophthora* species and environmental factors was analysed using R v4.2.1 [37] with logistic regressions using a quasibinomial distribution: environmental factors were considered as independent variables and the relative abundance as the response.

To study how *Phytophthora* species were grouped depending on their association with climate, we performed a hierarchical cluster using the estimated values of the logistic regressions with temperature and precipitation. The clustering was performed using JMP Pro v17.0.0 (SAS Institute Inc).

### Diversity analysis of *Phytophthora* communities

We studied associations between *Phytophthora* community diversity and climatic variables along the altitudinal and latitudinal gradients. In addition, associations between *Phytophthora* diversity and topography, tree diversity, and water chemistry were analysed along the altitudinal gradient. Only the diversity values of streams surveyed in the autumn along the altitudinal gradient were considered so as to be able to compare the values with those obtained for the latitudinal gradient survey. *Phytophthora* species belonging to clade 6 were provisionally classified as aquatic species and species belonging to clades 1, 2, 3, 7, 8, 10, or 11 were classified as terrestrial species [23, 38, 39]; however, some species were reclassified based on expert knowledge (T. Jung, personal communication). In an attempt to determine whether the climate effect on community assembly varied depending on the species residence time, we grouped species into two separate groups: “old” for species described >60 years ago and “new” for species described in the past 60 years (Supplementary Information Fig. S2). Several species whose description data were not representative of the time that had passed since they were first reported were re-assigned based on expert knowledge (T. Jung, personal communication). Aquatic species were not divided into old and new groups because only one of the eight aquatic species was classified as old. For analyses, reads were standardized by the total number of reads of each community using the total method of the decostand function of the “vegan” package [40] in R. For analyses in which aquatic, terrestrial, old terrestrial, and new terrestrial were considered separately, reads were standardized separately, e.g., reads of terrestrial species were not considered for standardizing reads of aquatic species. We calculated alpha diversity values using the “vegan” package. Species richness was calculated using the specnumber function. Shannon index was calculated with the diversity function. Evenness was calculated by dividing the Shannon diversity index by the natural log of species richness value. We calculated species richness, Shannon index, and evenness for communities that included all species and separately for communities that only included aquatic, terrestrial, old terrestrial, or new terrestrial species. Species richness and Shannon index were calculated for communities with one or more species, whereas evenness was calculated for communities with more than one species. To analyse the association between the diversity of *Phytophthora* communities and environmental factors, we built univariate linear models in R for each diversity parameter: environmental factors were considered as independent variables and the diversity parameter as the response.

To assess the level of spatial autocorrelation over the surveyed gradients, we fitted a mixed model using species diversity or functional diversity as the response, temperature as a fixed factor and both latitude and longitude as factors with a repeated spatial structure. The distribution of model residuals in a variogram was used to test whether the surveyed streams were spatially autocorrelated. No evidence of spatial autocorrelation was found, thus, we did not consider it in the analyses.

### Structural equation modelling and mediation analyses

The diversity of *Phytophthora* communities was correlated with several environmental factors; therefore, we used structural equation models (SEMs) and mediation tests to determine putative causal pathways between them. In the first SEM, we tested whether the diversity of *Phytophthora* communities was a result of an indirect effect of climate mediated by tree diversity. In the second SEM, we tested whether the link between temperature and *Phytophthora* diversity resulted in an indirect effect of temperature increasing the number of new species. In the third SEM, we tested whether *Phytophthora* community diversity was a result of an indirect effect of a warm and dry climate favouring drought-tolerant species. SEM analyses were performed with JMP Pro. To test indirect effects between variables, mediation analyses were conducted using the sem function of the package “lavaan” [41] in R.

### Functional diversity analysis

We analysed the association between the functional diversity of *Phytophthora* communities and climate along both the altitudinal and latitudinal gradients. Links between functional diversity and other environmental variables along the altitudinal gradient were tested as described for species diversity. Functional richness, functional evenness, and functional dispersion were calculated, taking into account only communities with more than two species, whereas community-level weighted mean (CWM) trait values were calculated for all communities. These functional parameters were calculated separately for all, aquatic, terrestrial, old terrestrial, and new terrestrial *Phytophthora* communities. Functional parameters and CWM trait values were calculated using the dbFD and functcomp functions of the “FD” package [42] in R, respectively. Functional traits for the 37 *Phytophthora* species detected, of which 34 are formally described and 3 are informally designated, were obtained from the trait database developed by Barwell et al. [28] and the tabular key and fact sheets for *Phytophthora* species from IDphy [29]. Missing data for some species were added based on literature searches. The functional traits selected for functional diversity analyses were related to morphological characteristics, environmental tolerance, and specialization (Supplementary Information Table S1). The association between functional diversity parameters and environmental factors was analysed in R using a univariate linear or a generalized linear model depending on the functional trait type (i.e., numeric, binary, or factor). The environmental factor was considered an independent variable and the functional parameter the response. Similarly, we evaluated the relationship between the dominant trait of the community obtained from CWM trait values and environmental factors.

### Seasonal analysis

To determine how the seasonal variation of *Phytophthora* was affected by summer or winter conditions, we compared species diversity, functional diversity, and CWM values of functional traits before and after summer and winter along the altitudinal gradient. We calculated species diversity, functional diversity, and CWM trait values independently for each sampling season (i.e., spring 2018, autumn 2018, spring 2019, and autumn 2019). To test the effect of summer, we built a mixed model using the season (i.e., autumn or spring) as a fixed effect and the year as a random effect and the diversity or CWM trait value parameter as the response. To test the effect of winter, we used data from autumn 2018 and spring 2019 samplings. We built a linear model using the season as an independent variable and the diversity or CWM trait value parameter as the response.

### Environmental filtering analysis

We searched for signs of environmental filtering and competitive exclusion by testing whether the functional richness of the community was lower than the null expectation for the observed species richness [30]. We simulated 5000 communities for each of the species richness values observed in our study by subsampling 15 samples without replacement [20]. A value of functional richness was considered significantly lower than the null expectation when it was below the lower 90% quantile of the simulated communities [30]. We repeated this process for communities with all species included, or with only aquatic, terrestrial, old terrestrial, or new terrestrial species included. We tested whether there was an association between environmental filtering and climatic variables using univariate linear models. In addition, to determine how much of the CWM value of a trait was explained by environmental filtering, we built univariate linear models in which the presence of environmental filtering was the independent variable and the CWM value of a trait was the response variable. These models were only built for *Phytophthora* communities that included all the species because the number of terrestrial and aquatic communities showing environmental filtering was too low for analysis.

## Results

### Sequencing output

The diversity of communities was adequately captured in both altitudinal and latitudinal gradients (Supplementary Information Fig. S3). In total, 42 OTUs were identified at the species level from 704 samples, of which 37 were identified as *Phytophthora* species, 4 as *Nothophytophthora* species, and 1 as a *Halophytophthora* species. The *Phytophthora* species belonged to clades 1, 2, 3, 6, 7, 8, 10, or 11. *Phytophthora* species were further classified as old (established >60 years ago) or new (established <60 years ago) depending on their putative residence time. Along both altitudinal and latitudinal gradients, the proportion of new species was higher than old species (58% vs 42% of the total *Phytophthora* reads along the altitudinal gradient, 60% vs 40% of the total *Phytophthora* reads along the latitudinal gradient). Along both gradients, reads of species with an aquatic lifestyle were more abundant than reads of terrestrial species. Aquatic species represented 71% and 86% of the total *Phytophthora* reads in the altitudinal and latitudinal gradient samples, respectively, whereas terrestrial species represented 29% and 14% of reads, respectively.

The most abundant aquatic and most abundant terrestrial species coincided along both altitudinal and latitudinal gradients: *Phytophthora lacustris* and *Phytophthora gonapodyides* were the most abundant aquatic species and *Phytophthora gallica* and *Phytophthora quercina* were amongst the most abundant terrestrial species (Supplementary Information Table S2).

### Biogeography of *Phytophthora* species

**Figure 2:**
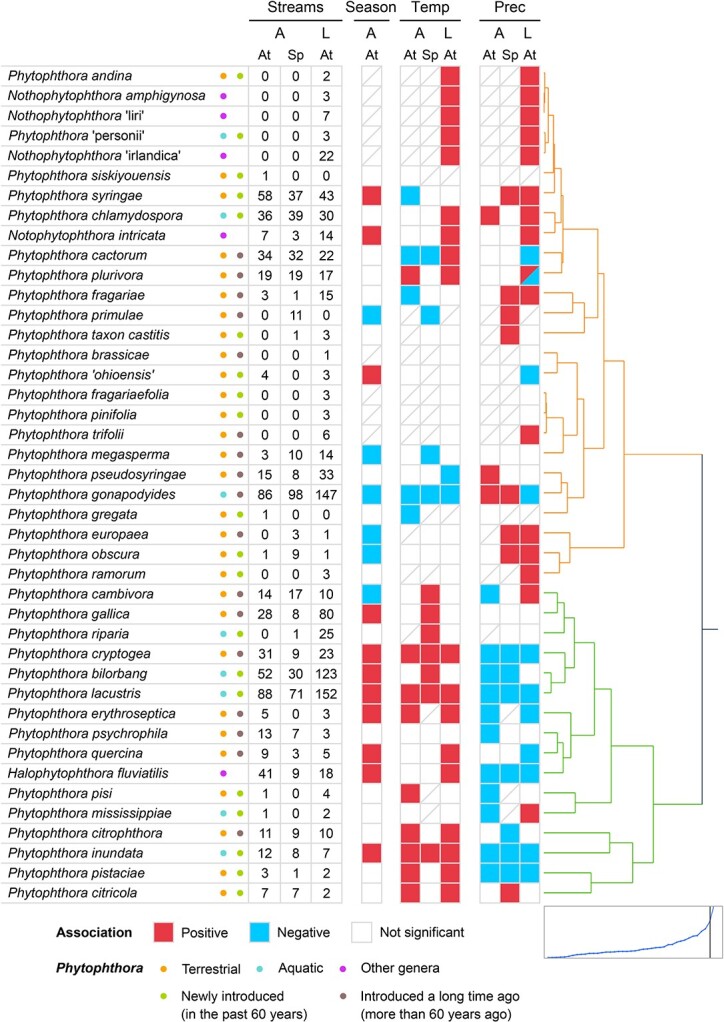
**Distribution of *Phytophthora* species based on environmental factors.** Association between the relative abundance of *Phytophthora* species and the season and environmental factors along both the altitudinal (A) and latitudinal (L) gradients. For the altitudinal gradient, the autumn (At) and spring (Sp) surveys were analysed separately. The values indicate the number of sites where a species was found. In total, 118 sites were sampled in the autumn and then again in spring along the altitudinal gradient, and 183 sites were sampled along the latitudinal gradient. Boxes with a diagonal line indicate that a species was not found along the gradient; quasibinomial models were not conducted for these species. Along the altitudinal gradient, the effect of the season on species abundance was tested by comparing the relative abundance between spring and autumn surveys. Red boxes show species more frequently observed in autumn than in spring whereas blue boxes show species more frequently found in spring. Along both the altitudinal and latitudinal gradients, the effect of temperature (Temp) and precipitation (Prec) on species abundance was tested using a logistic regression with a quasibinomial distribution. Annual and seasonal temperature and precipitation data were used for the analyses. Coloured boxes indicate that at least temperature or precipitation for one season or the annual average was significantly associated (*P* < 0.05) with species’ relative abundance. Red and blue indicate positive and negative associations, respectively. A box comprising both red and blue colours indicates that the association of the species with climatic variables was either positive or negative depending on the season (indistinctly of the season in which surveys were conducted). The species are clustered into two groups based on the association between the *Phytophthora* species distribution and climate. The distance graph of the clustering is shown bottom right.

**Figure 3:**
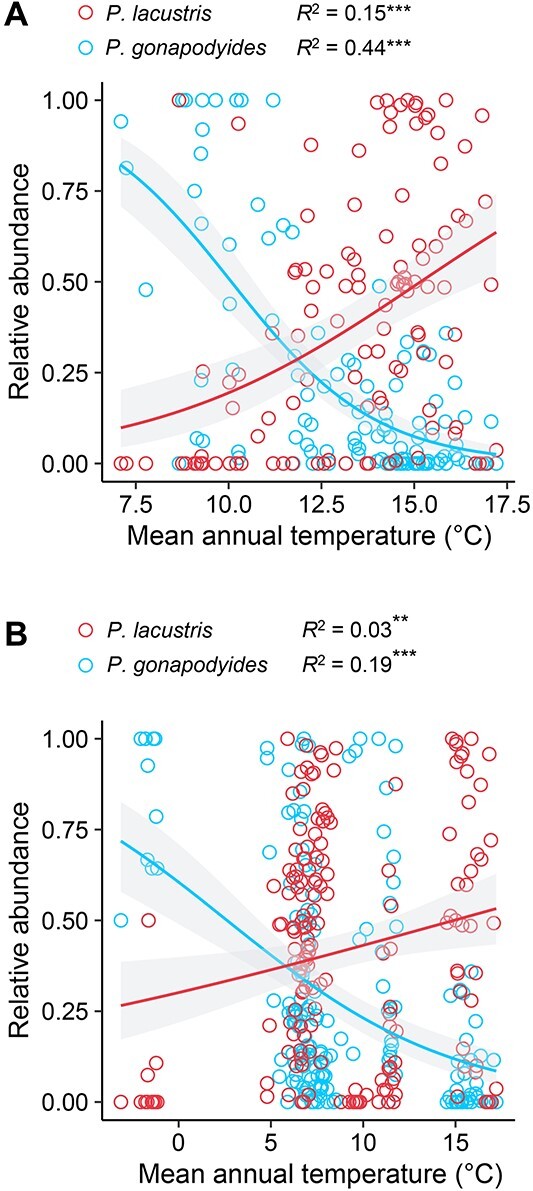
***Phytophthora lacustris* and *P. gonapodyides* distribution.** Relative abundance of *P. lacustris* and *P. gonapodyides* based on the mean annual temperature along (A) the altitudinal gradient and (B) the latitudinal gradient. The relationship between the relative abundance of species and temperature was analysed with logistic regressions using a quasibinomial distribution. McFadden’s *R*^2^ and *P* value are shown for each model. ^*^^*^ and ^*^^*^^*^ indicate significant associations at *P* < 0.01 and *P* < 0.0001, respectively.

We characterized the communities of *Phytophthora* plant pathogens in 263 stream sites distributed along a latitudinal gradient from southern to northern Europe and an altitudinal gradient spanning an elevation from 53 m.a.s.l to 1724 m.a.s.l in NE Spain. We found that the biogeography of *Phytophthora* species was associated with climate along both gradients. The majority of *Phytophthora* species showed a climatic signal along both the altitudinal and latitudinal gradients (77% and 79% of species were significantly associated with either temperature or precipitation during the autumn and spring surveys, respectively, along the altitudinal gradient, and 71% of species were significantly associated with either temperature or precipitation along the latitudinal gradient) (Fig. 2). Although *Phytophthora* distribution along the altitudinal gradient was also associated with other environmental factors, climate was a stronger predictor of species abundance than topography, tree diversity or water chemistry for 60% and 58% of the species during autumn and spring, respectively (Supplementary Information Tables S3 and S4). In general, individual *Phytophthora* species showed the same correlation with either temperature or precipitation along both gradients (43%, 46%, and 31% of species correlated with either temperature, precipitation, or both factors, respectively) (Fig. 2). This pattern was clearly illustrated by the two most abundant species *P. lacustris* and *P. gonapodyides*, which showed a positive and a negative association with temperature, respectively, along both gradients (Figs 2 and 3).

Based on their response to climatic variables, *Phytophthora* species were clustered into two groups, one that was associated with rainier conditions and the other with warm and dry conditions (Fig. 2). *Phytophthora* species associated with warm and dry conditions were more frequently detected in autumn than species associated with rainier conditions (*P* = 0.02) (Fig. 2). The distribution of sister genera *Halophytophthora* and *Nothophytophthora* mirrored preferences for either a dry climate or rain. *Halophytophthora fluviatilis* was found in streams in warm and dry regions, and was more frequently detected in autumn than in spring (Fig. 2). *Nothophytophthora* species were detected in warm and rainy regions (Fig. 2).

### Diversity and functional diversity of *Phytophthora* communities

**Figure 4:**
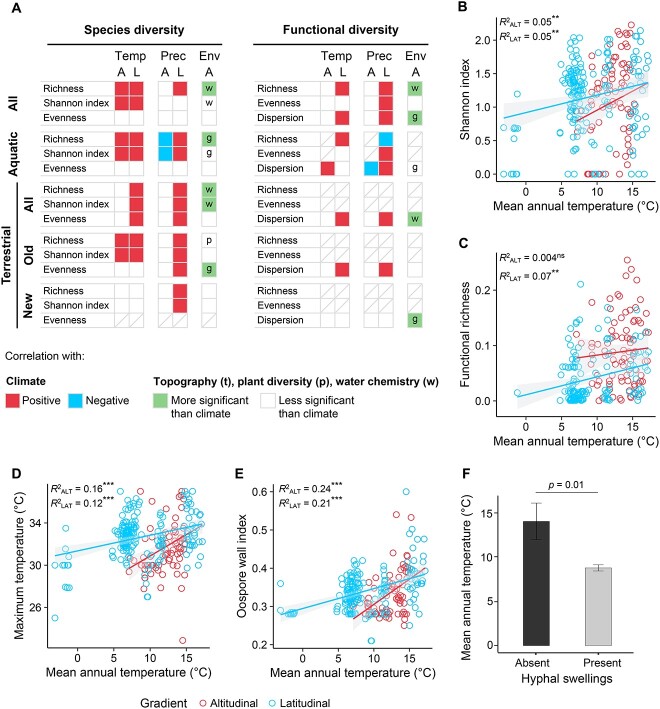
**Species diversity, functional diversity, and distribution of functional traits of *Phytophthora* communities along climatic gradients.** (A) Association of species diversity and functional diversity of *Phytophthora* communities with climate and other environmental (Env) factors (t, topography; p, plant diversity; and w, water chemistry) along an altitudinal (A) and a latitudinal (L) gradient. Association between diversity with climate and other environmental variables was tested using univariate linear models. Only data from autumn altitudinal gradient surveys (i.e. autumn 2018 and 2019) were used in the comparison with the latitudinal gradient survey data. In the “Env” column, the letters “t,” “p,” and “w” indicate the most significant environmental variable. A red or a blue box indicates a positive or negative estimated value of a significant association at *P* < 0.05 with at least temperature (Temp) or precipitation (Prec) for one season or for annual temperature or precipitation. A green box indicates that an environmental variable showed a more significant association (smaller *P* value) with diversity than any of the climatic variables. A box with a diagonal line indicates that an analysis was not conducted: >50% of streams did not have communities with the minimum number of species for calculating diversity indexes. (B, C) Variation of (B) the Shannon index and (C) the functional richness of *Phytophthora* communities with the mean annual temperature along both the altitudinal and latitudinal gradients. (D, E) Community-weighted mean (CWM) values of (D) the maximum temperature for growth and (E) the oospore wall index of *Phytophthora* species along the climatic gradients. Trait dominance was determined using the CWM values of traits. *R*^2^ value is shown for each model. ^*^^*^ and ^*^^*^^*^ indicate significant associations at *P* < 0.01 and *P* < 0.0001, respectively. “ns” indicates a non-significant correlation (*P* ≥ 0.05). (F) Distribution of *Phytophthora* communities dominated by species forming hyphal swellings along the climatic latitudinal gradient.

**Figure 5:**
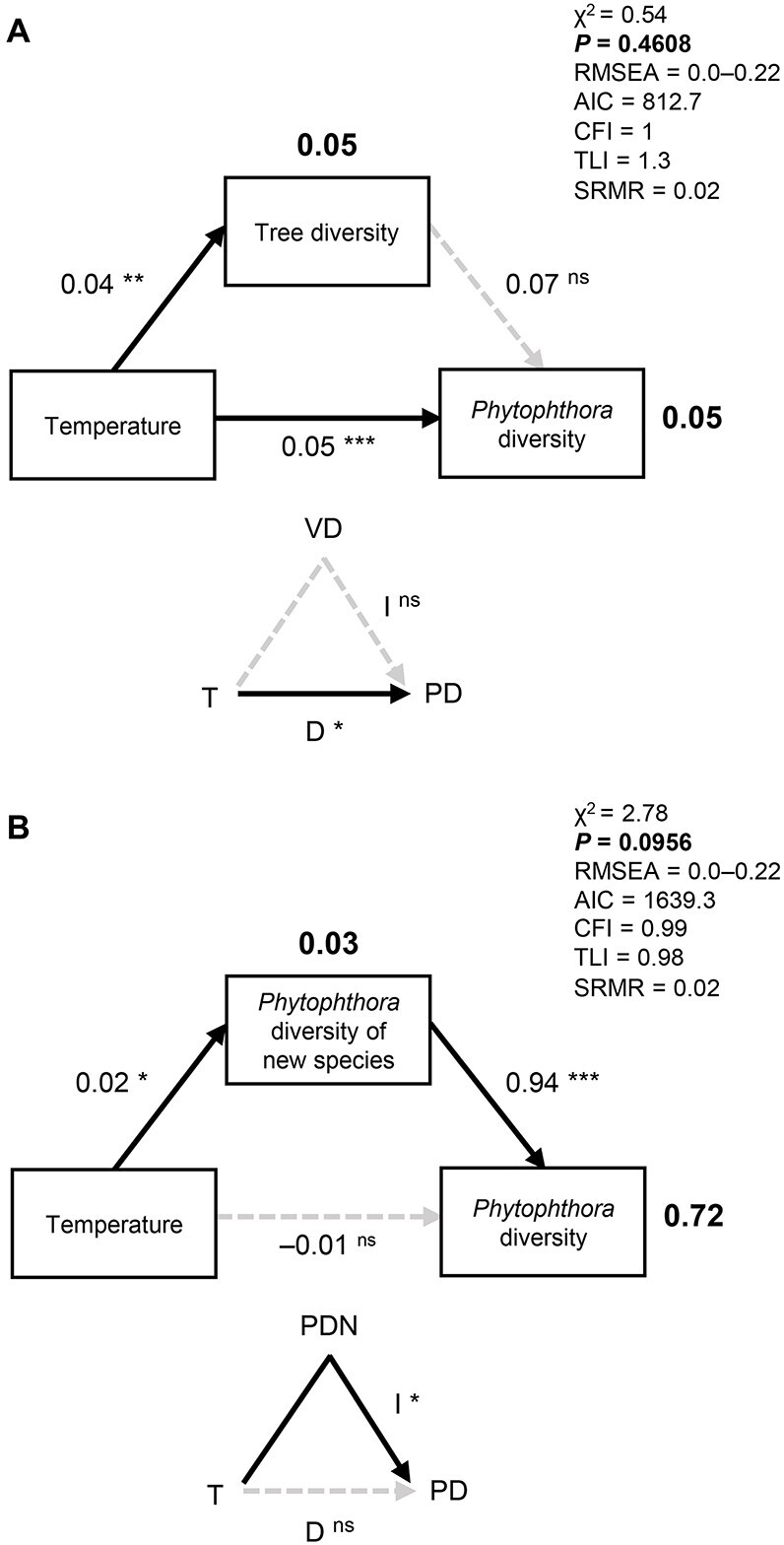
**Influence of temperature on *Phytophthora* community diversity.** (A, B) SEM analyses and mediation analyses of the influence of temperature on tree diversity and *Phytophthora* diversity along the altitudinal gradient (A), and influence of temperature on diversity of communities with recently established species or all species including data of both altitudinal and latitudinal gradients (B). Mediation analyses are shown in the small triangles, where direct (D) and indirect (I) effects of temperature are tested. *P* values > 0.05 indicate a better fit of the reduced SEM (only black-coloured pathways) over the full model including both black- and grey-coloured pathways. The chi-square value (χ^2^), the comparative fit index (CFI), the Tucker Lewis index (TLI), and the standardized root mean square residual (SRMR) of each model are shown. The Akaike information criterion (AIC) values comparing the final model (only pathways coloured in black) with the full model (both black- and grey-coloured pathways) are shown. The root mean square error of approximation (RMSEA) is expressed on its lower and upper limits at 90%. Values shown next to the pathways represent correlation estimates, whereas values shown in bold indicate the *R*^2^ of each variable. Grey dashed lines indicate non-significant associations (*P* ≥ 0.05). ^*^, ^*^^*^, and ^*^^*^^*^ indicate significant associations at *P* < 0.05, *P* < 0.01, and *P* < 0.001, respectively. “ns” indicates a non-significant correlation (*P* ≥ 0.05). T, annual mean temperature; VD, vegetation diversity; PD, *Phytophthora* diversity; PDN, *Phytophthora* diversity of new species.

Climate correlated with the diversity and functional diversity of *Phytophthora* communities along both the altitudinal and latitudinal gradients (Fig. 4A). Both species diversity and functional diversity increased with temperature and precipitation along the latitudinal gradient (Fig. 4A–C). Species diversity increased with increasing temperature and was also associated with other environmental variables, such as tree diversity or water chemistry along the altitudinal gradient (Fig. 4A and B). Nevertheless, mediation analysis supported a direct role of climate in *Phytophthora* diversity and ruled out an indirect effect of climate via tree diversity (i.e. an indirect effect of temperature on *Phytophthora* diversity via tree diversity was not significant) (Fig. 5A).

When considering newly established species separately from species that have been present in Europe for >60 years, a distinct pattern associated with climate was found. Although the species diversity of new species tended to be higher in areas with higher precipitation, the species diversity of old species was higher in warmer and rainy areas (Fig. 4A). Mediation analysis did not support a different role of climate in the contribution of new species and old species to the overall *Phytophthora* species pool. However, temperature increased *Phytophthora* diversity indirectly by favouring new species in warmer areas (Fig. 5B).

Mediation analysis also clarified how the *Phytophthora* species pool was related to climate in the driest and warmest areas of the altitudinal gradient. Under these conditions, the increase of diversity was mainly due to the increase of species belonging to the cluster adapted to warm and dry conditions (Supplementary Information Fig. S4).

### Distribution of functional traits of *Phytophthora* communities

Three key functional traits showed a common climatic signal along both gradients and across all types of *Phytophthora* species (i.e. aquatic and terrestrial): the optimum temperature for growth, the maximum temperature limit for growth, and the oospore wall index (Supplementary Information Fig. S5). Climate showed a stronger association with functional traits than other environmental variables along the altitudinal gradient (results not shown). In warm and dry areas, communities tended to be dominated by species with a high optimum temperature for growth, a high maximum temperature limit for growth, and a thick oospore wall (Fig. 4D and E, Supplementary Information Fig. S5). Species with hyphal swellings were significantly more frequent in cold areas along both gradients (Fig. 4F, Supplementary Information Fig. S5), whereas chlamydospores were more prevalent in warmer and drier climates (Supplementary Information Fig. S5).

Along both altitudinal and latitudinal gradients, communities in colder regions were dominated by species that had been described for a longer time than communities in warmer regions (Supplementary Information Fig. S5). However, even when controlling for residence time, traits related to environmental tolerance such as oospore wall thickness showed a common climatic signal along both gradients (Supplementary Information Fig. S6).

Other traits less directly related to the environmental tolerance such as host range were also associated with climate (Supplementary Information Fig. S5). In rainy regions, communities were dominated by species with caducous and papillate sporangia and with the capacity to cause foliar infections (Supplementary Information Fig. S5).

### Seasonality of *Phytophthora* communities

**Figure 6:**
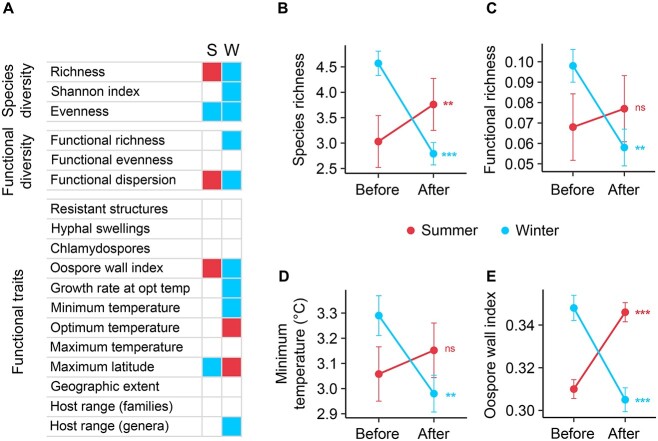
**Seasonality of *Phytophthora* communities.** (A) Variation of species diversity, functional diversity, and functional traits after summer (S) and winter (W). Univariate linear models were used to test associations. Coloured boxes indicate higher (red) or lower (blue) values of diversity or functional traits after summer (S) or after winter (W). (B–E) Comparison of (B) species richness, (C) functional richness, community-weighted mean values of (D) the minimum temperature limit for growth, and (E) the oospore wall index before and after summer and winter. ^*^^*^ and ^*^^*^^*^ indicate significant associations at *P* < 0.01 and *P* < 0.001, respectively. “ns” indicates a non-significant correlation (*P* ≥ 0.05).

The species diversity and functional diversity of *Phytophthora* communities varied after summer and after winter (Fig. 6A). An increase in species richness was detected after the summer (Fig. 6B). By contrast, both species richness and functional richness decreased after winter (Fig. 6B and C). Functional analysis revealed that species with a low minimum temperature for growth and a thin oospore wall dominated the community after winter (Fig. 6D and E). Furthermore, after winter, communities were dominated by species that have been found at higher latitudes (Fig. 6A). After the summer, communities became enriched with species with a thick oospore wall (Fig. 6E).

### Environmental filtering and competitive exclusion

**Figure 7:**
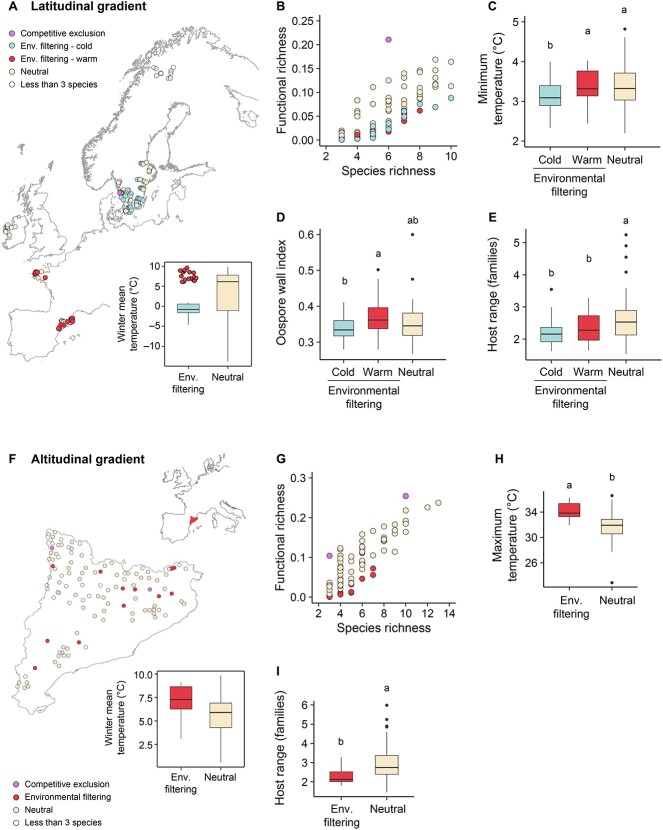
**Environmental filtering of *Phytophthora* communities.** (A, F) Distribution of plots showing environmental filtering along (A) the latitudinal gradient and (F) the altitudinal gradient. Inset boxplots show the winter mean temperature of plots with or without environmental filtering. (B, G) Association between functional richness and species richness for simulated communities along (B) the latitudinal gradient and (G) the altitudinal gradient. (C–E) CWM value of (C) the minimum temperature limit for growth, (D) the oospore wall index, and (E) the host range of *Phytophthora* communities located in areas with or without environmental filtering along the latitudinal gradient. (H, I) CWM value of (H) the maximum temperature limit for growth and (I) the host range of *Phytophthora* communities located in areas with or without environmental filtering along the altitudinal gradient. Different letters indicate statistically significant differences between values (*P* < 0.05).

Environmental filtering was observed far more frequently than competitive exclusion (14% vs 2% of sites along the altitudinal gradient and 60% vs 0.7% of sites along the latitudinal gradient) (Fig. 7B and G) and was more prevalent along the latitudinal gradient than along the altitudinal gradient (*P* = 0.0001). Environmental filtering was observed at both extremes of the latitudinal gradient, in regions with low winter temperatures and in regions with a hot and dry climate (Fig. 7A). At northern latitudes, communities with signs of environmental filtering lost species adapted to mild winters and were enriched with species adapted to low minimum temperatures (Fig. 7C). At southern latitudes, environmental filtering eliminated species with thin oospore walls and only those with thick oospore walls survived (Fig. 7D). At both northern and southern latitudes, environmental filtering decreased the frequency of species with a wide-host range, with communities becoming more dominated by host-specific species (Fig. 7E). Environmental filtering was observed in warm regions along the altitudinal gradient (Fig. 7F). In these regions, communities became dominated by species with a high maximum temperature limit for growth (Fig. 7G). Similar to the situation observed along the latitudinal gradient, environmental filtering reduced the host range (Fig. 7I).

## Discussion

Understanding the role of climate in the distribution of plant pathogens is paramount owing to the exacerbating effects of climate change. Although this has been studied for communities of other plant-associated microbes, such as bacteria and mycorrhizal and endophytic fungi [3–7], less is known about the effect of climate on the assembly of plant pathogen communities. Here, we describe how diversity and functional diversity of *Phytophthora* communities varied seasonally along a climatic gradient in Europe.

Along the latitudinal gradient from Mediterranean to boreal conditions, signs of environmental filtering caused by climate were much more common than signs of competitive exclusion. At northern latitudes, the functional diversity of *Phytophthora* communities was constrained in areas with the lowest winter temperatures. Species unable to survive low temperatures showed a lower frequency whereas species with low minimum temperature limits for growth showed a higher frequency. Moreover, species with the ability to form hyphal swellings became dominant. Survival structures have been associated with the ability of *Phytophthora* to tolerate low temperatures [8, 20]. Coping with cold seems to be the main constraint for *Phytophthora* at northern latitudes, thus, given the predicted climate change, range expansions in these regions can be predicted in the future.

At southern latitudes, and regardless of a greater tree diversity, species diversity increased with temperature. However, in areas where warmer conditions were not matched with rainfall increases, functional diversity was lower and signs of environmental filtering associated with a drier climate were observed. In these areas, species with a thin oospore wall and that were unable to grow under high temperatures were filtered out and species adapted to drought became dominant. Response traits related to climatic requirements, such as the optimum temperature for growth, had been previously found to be key traits predicting the invasion success of pathogenic fungi [43]. Similarly, oospore thickness has been associated with greater desiccation resistance and long-term survival [24–26]. The role of drought as an environmental filter has been observed in other organisms, such as alpine plants, with their differentiation in terms of drought tolerance mirroring their geographic distribution [44], and aquatic fungal communities, whose functional diversity was reduced at ephemeral sites compared with that of permanent watercourses [45]. Thus, not unexpectedly, coping with a hot and dry climate appears to be a key trait for *Phytophthora* species to establish at southern latitudes.

Even though environmental filtering dominated, we also found some sites showing competitive exclusion along both gradients. Although the number of sites showing competitive exclusion was too low to characterize, we speculate that warm and wet areas such as rainforests could perhaps provide the right conditions for such a phenomenon. Traits related to different infection strategies, such as caducous sporangia, or new infection courts, such as the capacity to infect foliage, seemed to co-exist with others in the rainier parts of Atlantic Europe. Furthermore, *Nothophytophthora*, a sister genus of *Phytophthora*, seems to be prevalent in these areas, perhaps illustrating a putatively higher diversity of niches and opportunities. The co-existence of wide and narrow host strategies in areas with no signs of environmental filtering, as opposed to the presence of host-specific species in northern and southern Europe, also suggests the relevance of traits unrelated to environmental stress in warm and rainy areas. Some studies have reported lower levels of *Phytophthora* species diversity in tropical lowland rainforests than in tropical montane forests [46, 47]. However, whether the functional diversity of rainforests is also lower than expected remains to be seen. Thus, further surveys are required to confirm this hypothesis.

The species diversity and functional diversity of *Phytophthora* communities varied between seasons. Seasonal species variation following winter and summer mirrored the observed patterns at a latitudinal and an altitudinal level. After winter, both species diversity and functional diversity decreased and, as in the northern parts of Europe, species with low minimum temperature limits for growth became dominant. After summer, an increase in species richness was detected and, as in southern Europe, communities became dominated by species with a thick oospore wall. Seasonal changes therefore also reflect the role of climatic conditions in shaping microbial communities regardless of their pathogenic potential for plants.

Biogeography and the assembly of plant pathogen communities can be altered by introduced species. Along both the altitudinal and the latitudinal gradients, we found that communities located in warmer regions tended to be dominated by species introduced more recently. This pattern could suggest that diversity differences along a climatic gradient could simply be a reflection of residence time [48] and that it is only a matter of time before introduced species spread and establish in more northerly and colder regions. However, it is also likely that most imports of infected plant material have come from warmer regions outside Europe, resulting in the introduction of mostly thermophilic rather than cold-tolerant species. Regardless of residence time, traits related to environmental tolerance, such as oospore wall thickness, showed a climatic signal and were predictors of biogeography along both altitudinal and the latitudinal gradients, indicating that range expansions are unlikely without proper adaptations. However, with climate change, it is likely that many of these *Phytophthora* species will be able to survive and establish at more northern latitudes.

We should note that the climatic signal in this study is indirectly obtained from a geographic, topographic, and seasonal distribution of communities, and therefore the role of climate was not tested per se. However, the coincidence in the signal and the support found from different functional adaptations or response traits leaves little room for alternative explanations for the observed patterns.

In conclusion, our analyses indicate that climate poses a significant constraint on the distribution of *Phytophthora* species in Europe. Two key processes determined the assembly of *Phytophthora* species communities. At northern latitudes, the cold climate restricted species richness and acted as a filter for *Phytophthora* distribution, selecting species with a low minimum temperature limit for growth. By contrast, at southern latitudes, a dry climate posed a strong environmental filter for *Phytophthora* communities. This resulted in low functional diversity owing to the dominance of drought-tolerant *Phytophthora* species with thick oospore walls, a high optimum temperature for growth, and a high maximum temperature limit for growth. The ability to cope with dry conditions seems to be key to colonizing southern latitudes whereas the ability to survive under cold conditions seems to be key for colonizing northern latitudes. Our analyses highlight the role played by climate in the distribution of pathogens besides their parasitic lifestyle and may explain why native and exotic pathogens often do not completely cover the range of their hosts [49].

## Supplementary Material

Supplementary_information_final_wrae010

Supplementary_Tables_S3_S4_final_wrae010

## Data Availability

The datasets generated during the current study are available in the figshare repository, https://doi.org/10.6084/m9.figshare.24559555 [50].
