# Supplementary material for: Climate acts as an environmental filter to plant pathogens
Source: ISME J. 2024 Jan 23;18(1):wrae010. doi: 10.1093/ismejo/wrae010 (PMC10926774; doi:10.1093/ismejo/wrae010)
Supplement: Supplementary_information_final_wrae010 [file supplementary_information_final_wrae010.pdf]

Supplementary information

Methods S1. Stream surveys and determination of water chemistry parameters.

Methods S2. Obtention of climatic and vegetation data.

Figs. S1. Location of the surveyed streams of the altitudinal gradient.

Fig. S2. Density of *Phytophthora* species based on their residence time.

Fig. S3. Rarefaction and species accumulation curves.

Fig. S4. Influence of climatic variables on *Phytophthora* diversity of drought-tolerant species.

Fig. S5. Association between functional traits of *Phytophthora* communities and climate.

Fig. S6. Distribution of functional traits of *Phytophthora* communities of “old” terrestrial species along the climatic gradients.

Table S1. Functional traits of *Phytophthora* used for functional diversity analyses.

Table S2. *Phytophthora* species detected along the altitudinal and latitudinal gradients.

Table S3. Association between *Phytophthora* species during autumn and environmental factors.

Table S4. Association between *Phytophthora* species during spring and environmental factors.

Methods S1. Stream surveys and determination of water chemistry parameters.

We surveyed a total of 263 streams along two gradients, i.e., altitudinal and latitudinal (Fig. 1a). For the altitudinal gradient survey, 118 streams from independent watersheds were sampled in Catalonia (NE Spain) in the spring and autumn in 2018 and 2019 (Supplementary Information Fig. S1). The latitudinal gradient survey comprised 183 stream sites ranging from NE Spain to northern Sweden: 21 streams were surveyed in Brittany (NW France) between June and August 2018; 15 streams were surveyed in Ireland during the spring months of 2020; 16 rivers in southern Sweden were each sampled at six different sites (with an average distance of ca. 20 km between sites) along their catchments (i.e., 96 sites in total) between August and October 2013, and again in 2014; 13 streams were surveyed in northern Sweden during August 2017; and 38 of the streams that were sampled as part of the altitudinal gradient survey in Spain in the autumn were also included in the latitudinal survey. These 38 streams were at an elevation of <479 m above sea level (m.a.s.l.), equivalent to the elevation of the highest site in the latitudinal gradient survey. The altitudinal gradient covered a stream network ranging from 53 to 1724 m.a.s.l., covering a range of climatic conditions (with mean annual temperatures ranging from 7.1 to 17.2°C and total annual precipitation ranging from 388 to 1180 mm) (Fig. 1d). The latitudinal gradient ranged from 40.9 to 68.4°N, covering areas with mean annual temperatures ranging from –3.1 to 17.2°C and total precipitation ranging from 388 to 1549 mm (Fig. 1d). Although no correlation between temperature and precipitation was found along the latitudinal gradient (Fig. 1b), temperature and precipitation were negatively correlated along the altitudinal gradient (Fig. 1c). Sampling locations were chosen on the basis that they had an upstream catchment area that was mainly forest with a minimal presence of agricultural land or urban areas and that they were easily accessible.

Sampling was carried out by collecting 6 l of water at each site, which were subsequently filtered through an 8-µm membrane (Merck Millipore, Cork, Ireland) attached to the pump of

an agricultural hand sprayer with a polysulfone filter holder [1]. Samples collected in France were filtered through a 5-µm membrane. Membranes were replaced every time they became obstructed until the entire 6 l sample had been filtered. Membranes were stored in Petri dishes at 5°C before transportation to the laboratory and then stored at −20°C until processed for DNA extraction. Pumps were rinsed with 5% sodium hypochlorite and distilled water between sampling sites to avoid cross-contamination.

During the altitudinal gradient survey in the spring of 2019, water samples were collected at each sampling site in 60-ml syringes. These samples were filtered through pre-combusted (450°C) GF/F filters (Whatman, Maidstone, UK) and stored in pre-combusted 42-ml glass vials at 4°C. Samples were analysed by the Catalan Institute for Water Research (ICRA) to determine the following parameters: the concentration of dissolved organic carbon (DOC) in samples acidified to pH 2–3 and using a Shimadzu TOC-V CSH analyser (Japan), the specific ultraviolet absorbance at 254 nm [2] ($SUVA_{254}$) using an Agilent 8453 diode array spectrophotometer, and the fluorescence index (FI), humidification index (HIX) and specific fluorescence at various peaks (A, C, M, T, B), which were obtained using an F-7000 spectrofluorometer (Hitachi, Japan) according to previously described procedures [3].

1. Redondo MA, Boberg J, Stenlid J, Oliva J. Contrasting distribution patterns between aquatic and terrestrial *Phytophthora* species along a climatic gradient are linked to functional traits. *ISME J* 2018; **12**: 2967–2980.

2. Weishaar JL, Aiken GR, Bergamaschi BA, Fram MS, Fujii R, Mopper K. Evaluation of specific ultraviolet absorbance as an indicator of the chemical composition and reactivity of dissolved organic carbon. *Environ Sci Technol* 2003; **37**: 4702–4708.

3. Catalán N, Pastor A, Borrego CM, Casas-Ruiz JP, Hawkes JA, Gutiérrez C, et al. The relevance of environment vs. composition on dissolved organic matter degradation in freshwaters. *Limnol Oceanogr* 2021; **66**: 306–320.

Methods S2. Obtention of climatic and vegetation data.

Climatic data for the altitudinal gradient sampling plots were obtained from the Meteorological Service of Catalonia website (https://www.meteo.cat). These data were processed with the “meteoland” package [4] in R to obtain monthly mean temperature and precipitation values for 1976 to 2020. Climatic data for the latitudinal gradient sampling plots (i.e., plots in France, Ireland and Sweden) were obtained from the KNMI Climate Explorer webpage (https://climexp.knmi.nl/start.cgi). Monthly climatic data (mean temperature and precipitation) were obtained from the E-OBS 0.25° gridded dataset [5] for 1975 to 2021. For sampling plots of the altitudinal and latitudinal gradient surveys, we computed the average temperature and precipitation for each month over the available period as well as the average annual temperature and total annual precipitation. Mean temperature and precipitation values for winter, spring, summer and autumn were calculated using the mean monthly temperature or precipitation values for December to February, March to May, June to August and September to November, respectively.

For each stream of the altitudinal gradient survey, tree diversity was computed with the Shannon index for the riverbank vegetation and the Shannon index for the watershed vegetation. For each watershed, we calculated the following topographical parameters: the proportion of each type of land cover (i.e., urban, agricultural or forest), the total watershed area, aspect and slope. The proportion of each tree species in the watershed was determined using ESRI GIS ArcMap (version 10.7)[6]. Topographical variables were obtained from a 5 × 5 m terrain elevation model produced by the Cartographic and Geological Institute of Catalonia (ICGC). Land cover data were obtained from the System of Information on Spain’s Land Use (SIOSE). Tree species data were obtained from the Spanish Forest Map 1:25 000. Shannon index for the watershed vegetation was based on the species (%) data obtained from

the Spanish Forest Map, and the Shannon index for the riverbank vegetation was based on the visually estimated riverbank vegetation species (%) data. Altogether with the Shannon indices, the presence (%) of the most dominant tree genera in the watershed (i.e., *Pinus*, *Quercus*, *Fagus*, *Betula*, *Abies*, *Castanea*, *Juniperus* and *Corylus*) and on the riverbank (i.e., *Populus* and *Alnus*) were used as tree variables.

4. De Cáceres M, Martin-StPaul N, Turco M, Cabon A, Granda V. Estimating daily meteorological data and downscaling climate models over landscapes. *Environ Model Softw* 2018; **108**: 186–196.

5. Cornes RC, van der Schrier G, van den Besselaar EJM, Jones PD. An ensemble version of the E-OBS temperature and precipitation data sets. *J Geophys Res Atmos* 2018; **123**: 9391–9409.

6. Environmental Systems Research Institute. ArcGIS Desktop: Release 10. 2018. Redlands, CA.

Fig. S1. Location of the surveyed streams of the altitudinal gradient. a,b, Location of (**a**) the surveyed streams (red-coloured points) and (**b**) the independent watersheds where streams were located (green-coloured polygons). **c,d,** Orthophoto and (**d**) topographic map showing the physical delimitation of the watersheds (rivers are shown in blue, the delimitation of the watershed is shown in red, and the surveyed location is shown with a red-coloured point).

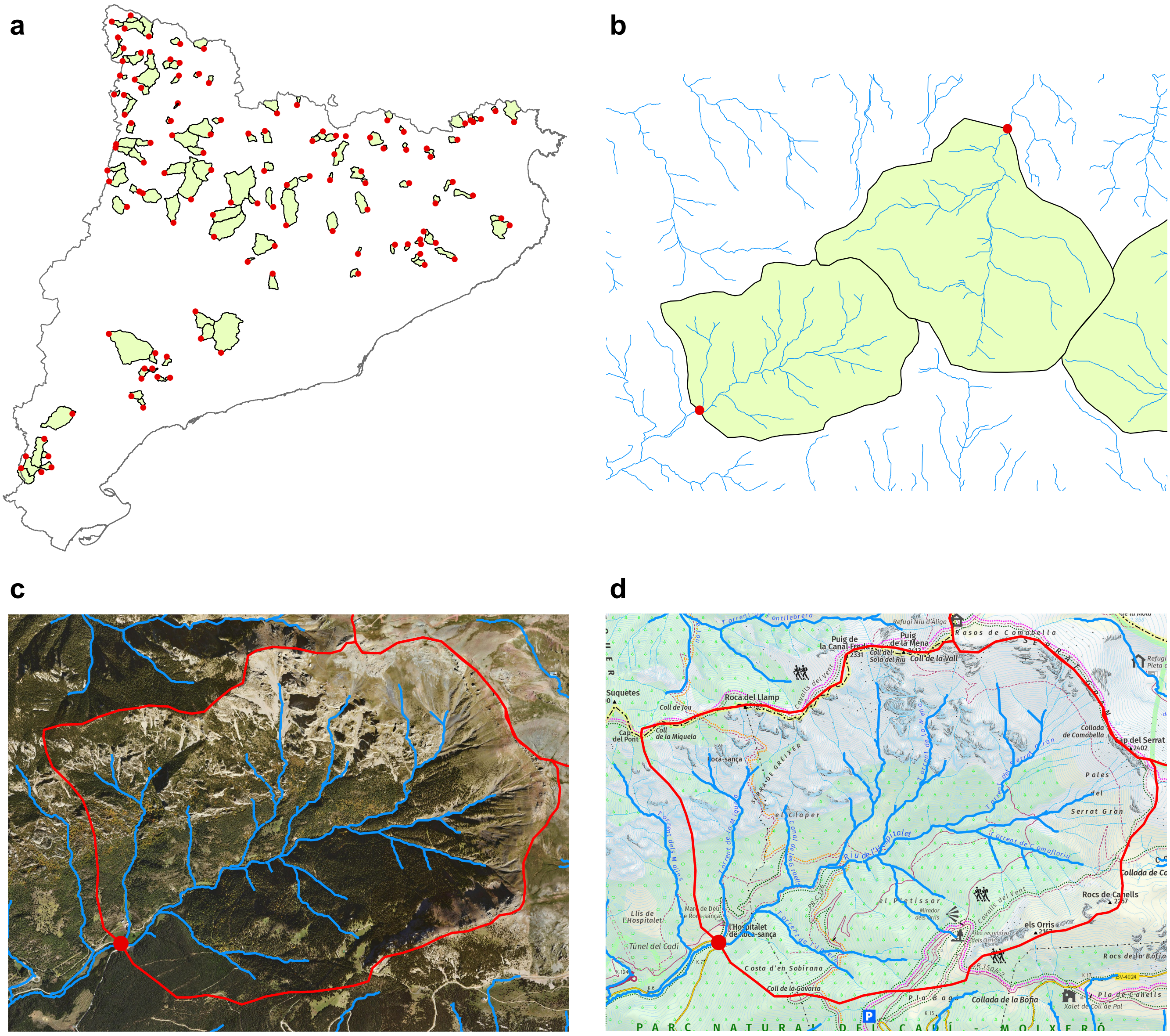

Fig. S2. Density of *Phytophthora* species based on their residence time. "Old species" refers to species first described more than 60 years ago and "new species" refers to species described in the past 60 years.

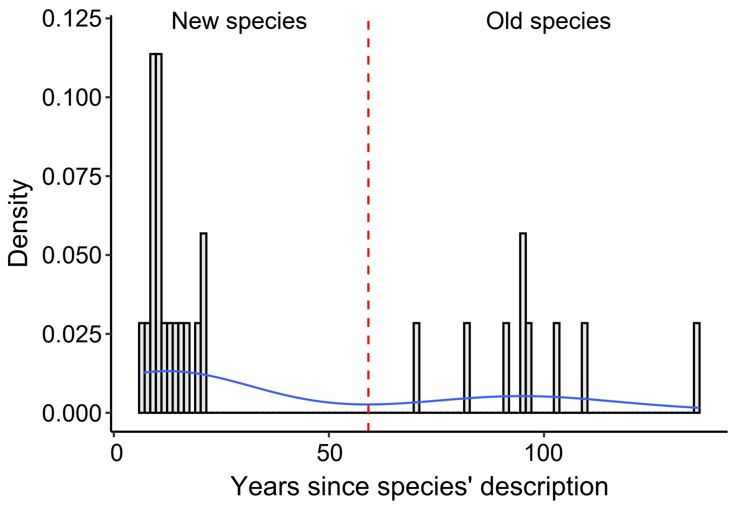

Fig. S3. Rarefaction and species accumulation curves. a,b,c, Rarefaction curves for (**a**) the autumn survey and (**b**) the spring survey in the altitudinal gradient and (**c**) the latitudinal gradient. **d,e,f,** Species accumulation curves for (**d**) the autumn survey and (**e**) the spring survey in the altitudinal gradient and (**f**) the latitudinal gradient.

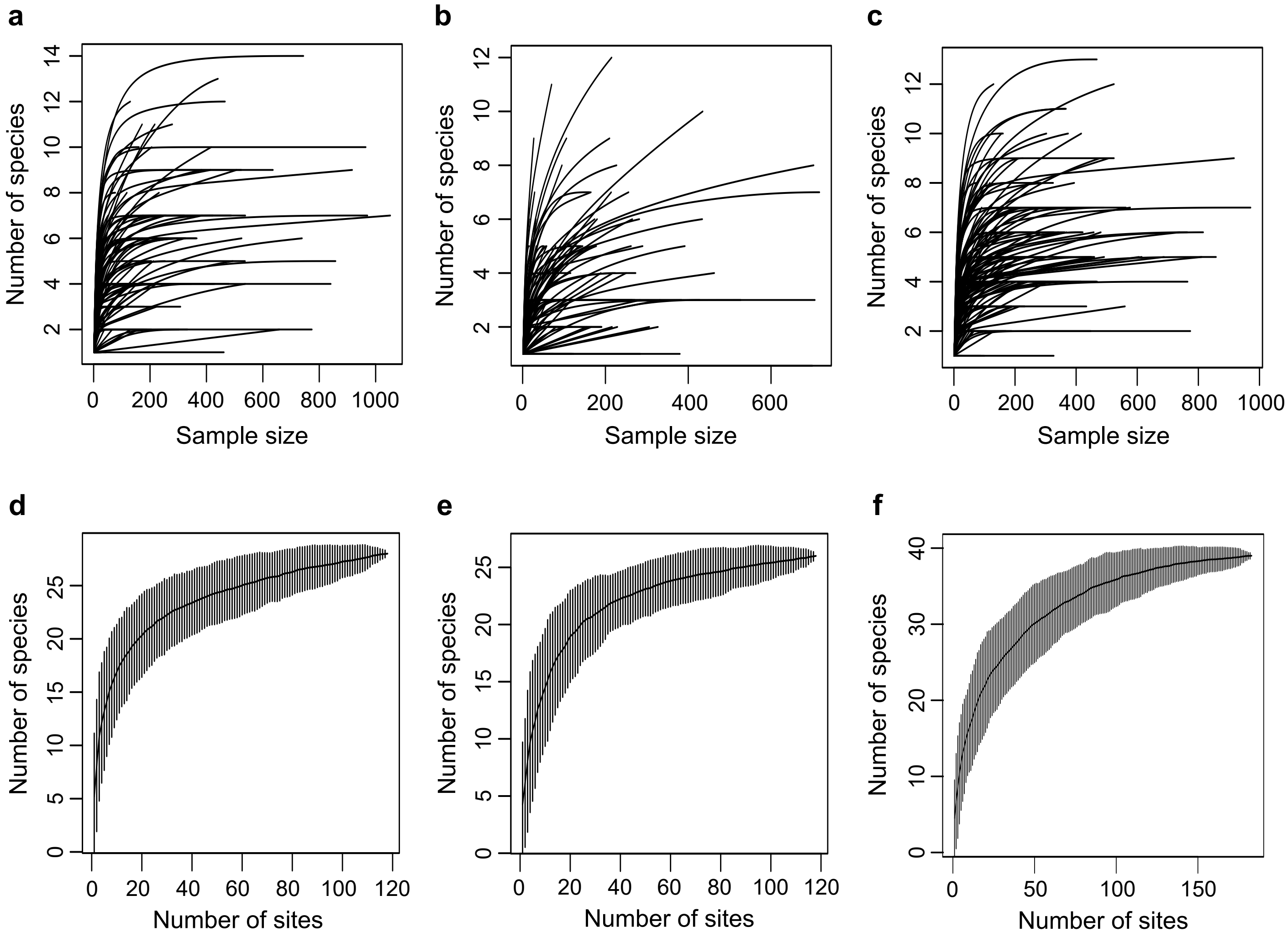

Fig. S4. Influence of climatic variables on *Phytophthora* diversity of drought-tolerant species. Structural equation model (SEM) analysis and mediation analysis of the influence of a warm and dry climate on *Phytophthora* diversity of drought-tolerant species along the altitudinal gradient. The chi-square value (χ^2), the Comparative Fit Index (CFI), the Tucker Lewis index (TLI) and the Standardized Root Mean Square Residual (SRMR) of the model are shown. The root mean square error of approximation (RMSEA) is expressed on its lower and upper limits at 90%. The circle indicates a latent variable. Values shown next to the pathways represent correlation estimates and the R^2 of each variable is shown in bold. * and *** indicate significant associations at $p < 0.05$ and $p < 0.001$, respectively. "ns" indicates a non-significant correlation ($p \geq 0.05$). Mediation analysis is shown in the small triangle, where direct (D) and indirect (I) effects of climate are tested. C, climate; PD, *Phytophthora* diversity; PDD, *Phytophthora* diversity of drought-tolerant species.

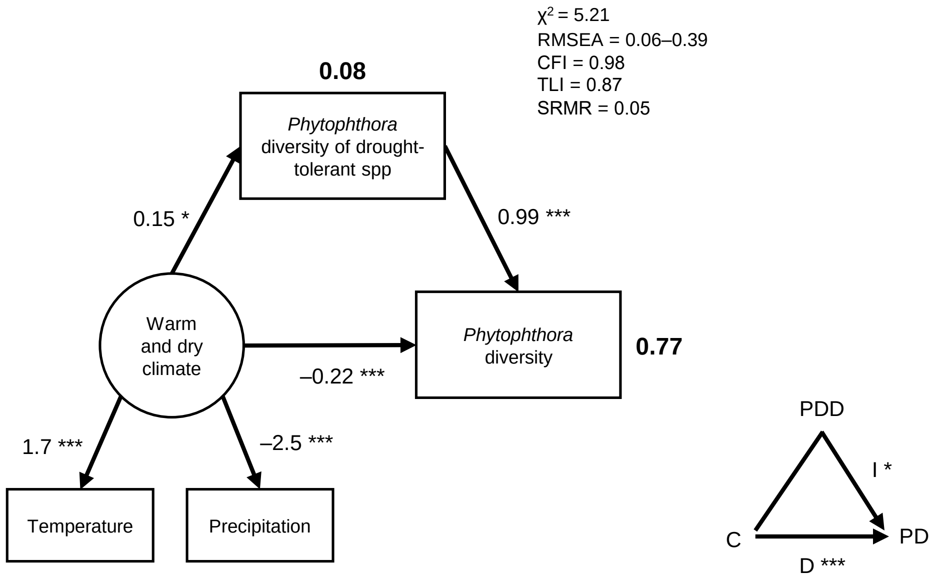

Fig. S5. Association between functional traits of *Phytophthora* communities and climate.

Association between functional traits of *Phytophthora* communities and climatic factors along both the altitudinal (A) and latitudinal (L) gradients. Boxes with a diagonal line indicate analyses that were not conducted because the dominant trait was the same for all communities. Green boxes represent a significant association of climate at $p < 0.05$ with a categorical functional trait. Red and blue boxes indicate a positive or negative estimated value of a significant association at $p < 0.05$ with at least temperature or precipitation for one season or for annual temperature or precipitation. Boxes comprising both red and blue colours indicate that the association of functional traits with climatic variables was either positive or negative depending on the season.

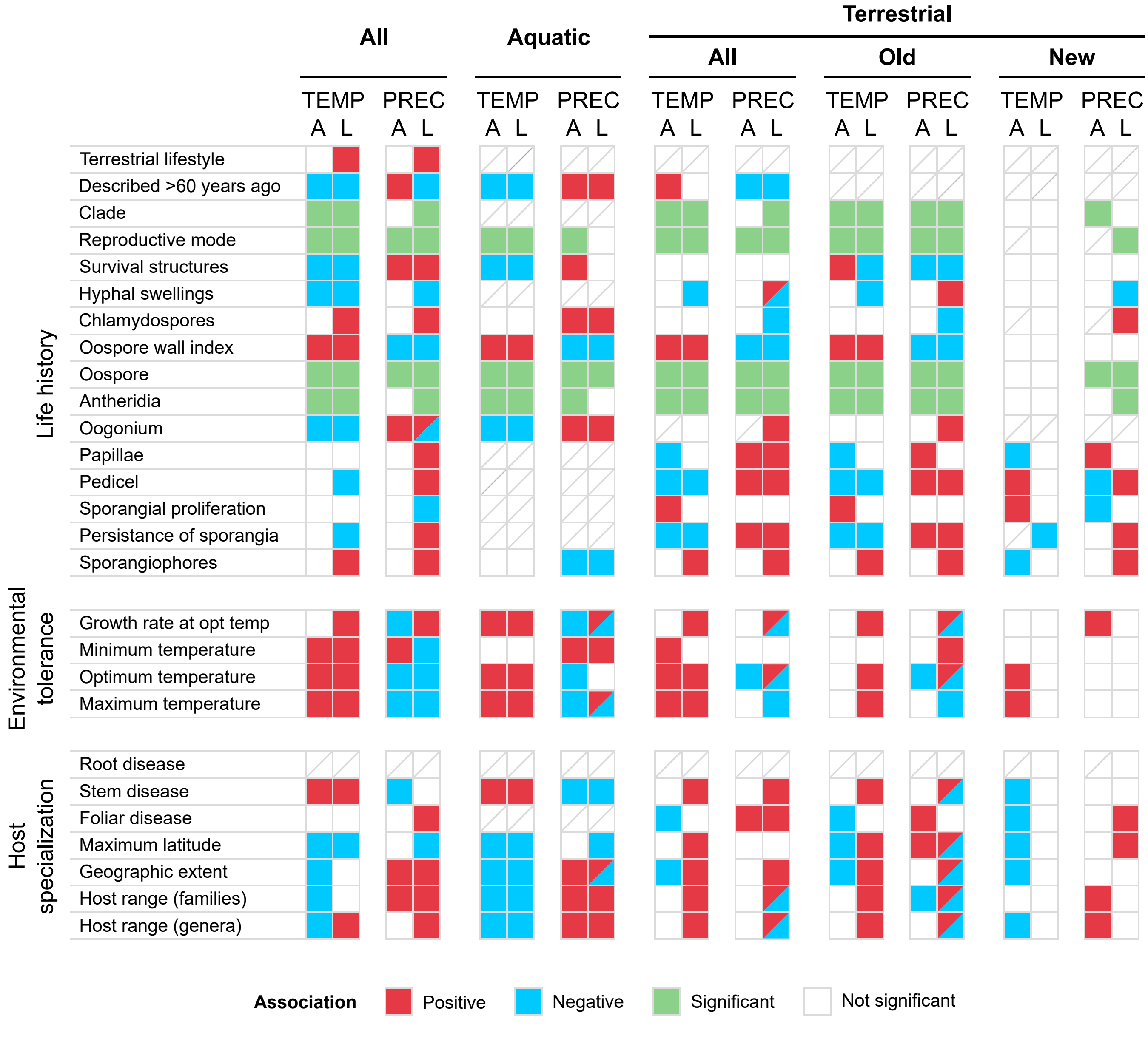

Fig. S6. Distribution of functional traits of *Phytophthora* communities of old terrestrial species along the climatic gradients. Community-weighted mean values of the oospore wall index of old species (first described more than 60 years ago) along the climatic gradients. R^2 and p value are shown for each model. ** and *** indicate significant associations at $p < 0.01$ and $p < 0.001$, respectively.

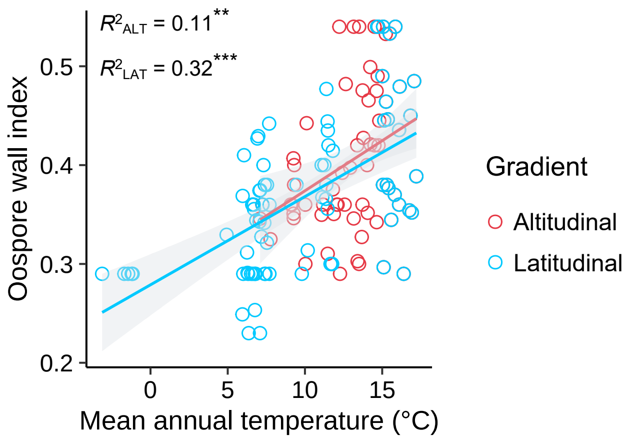

Table S1. **Functional traits of *Phytophthora* used for functional diversity analyses.**

Trait	Type	Trait levels
**Life history**		
Lifestyle	Factor	A (aquatic); B (terrestrial)
Time since description	Factor	A (new); B (old)
Clade	Factor	A (Clade 1); B (Clade 2); C (Clade 3); D (Clade 6); E (Clade 7); F (Clade 8); G (Clade 10); H (Clade 11)
Reproductive mode	Factor	A (homothallic); B (heterothallic); C (sterile)
Survival structures	Binary	0 (absent); 1 (present)
Hyphal swellings	Binary	0 (absent); 1 (occasionally and present)
Chlamydospores	Binary	0 (absent); 1 (occasionally and present)
Oospore wall index	Numeric	
Oospore	Factor	A (plerotic); B (aplerotic); C (both)
Antheridia	Factor	A (paragynous); B (amphigynous); C (both)
Oogonium	Binary	0 (smooth); 1 (ornamented)
Papillae	Binary	0 (non-papillate); 1 (semi-papillate and papillate)
Pedicel	Binary	0 (no pedicel); 1 (short, medium or long pedicel)
Sporangial proliferation	Binary	0 (non-proliferating); 1 (proliferating)
Persistence of sporangia	Binary	0 (persistent); 1 (occasionally caducous and caducous)
Sporangiophores	Binary	0 (unbranched); 1 (branched)
**Environmental tolerance**		
Growth rate at optimum temperature	Numeric	
Minimum temperature limit for growth	Numeric	
Optimum temperature for growth	Numeric	
Maximum temperature limit for growth	Numeric	
**Specialization**		
Root disease	Binary	0 (root disease not reported); 1 (root disease reported)
Stem disease	Binary	0 (stem disease not reported); 1 (stem disease reported)
Foliar disease	Binary	0 (foliar disease not reported); 1 (foliar disease reported)
Maximum latitude	Numeric	
Geographic extent (countries reached)	Numeric	
Host range (families)	Numeric	
Host range (genera)	Numeric	

Table S2. *Phytophthora* species detected along the altitudinal and latitudinal gradients. Operational taxonomic units identified at the species level and proportion of reads (%) and their 95% confidence intervals (CI) along both the altitudinal and latitudinal gradients. Residence time: old indicates species that were first described > 60 years ago; new indicates species that were described < 60 years ago.

	**Altitudinal gradient (*n* = 118)**				**Latitudinal gradient (*n* = 183)**	
	Autumn		Spring			
	Median	CI	Median	CI	Median	CI
**Aquatic species**						
P. bilorbang	7.5	3.9–13.9	3.2	1.2–8.5	10.3	6.7–15.6
P. chlamydospora	1.8	0.5–6.6	3.0	1.0–8.1	1.2	0.3–4.4
P. gonapodyides	21.3	14.8–29.6	45.6	36.9–54.7	25.4	19.6–32.2
P. inundata	1.1	0.2–5.9	0.3	0.01–7.2	0.6	0.1–3.8
P. lacustris	33.0	25.2–42.0	25.9	18.8–34.6	35.8	29.2–43.0
P. mississippiae	0.001	0.0–100.0	–	–	0.03	0.0–49.7
P. 'personii'	–	–	–	–	0.01	0.0–97.7
P. riparia	–	–	0.008	0.0–99.9	0.4	0.04–3.8
**Terrestrial species**						
**Old**						
P. brassicae	–	–	–	–	0.002	0.0–100.0
P. cactorum	3.3	1.2–8.5	3.4	1.3–8.6	0.5	0.05–3.8
P. cambivora	0.3	0.01–7.9	1.2	0.2–6.0	0.2	0.005–5.4
P. citrophthora	1.3	0.3–6.1	1.6	0.4–6.5	0.9	0.2–4.1
P. cryptogea	2.5	0.8–7.6	0.1	0.001–14.1	0.5	0.07–3.8
P. erythroseptica	0.2	0.003–10.6	–	–	0.03	0.0–55.7
P. europaea	–	–	0.04	0.0–79.6	0.01	0.0–98.5
P. fragariae	0.1	0.0–22.8	0.3	0.01–8.1	0.1	0.003–6.5
P. gallica	4.5	1.9–10.1	0.5	0.03–6.2	3.8	1.8–7.8
P. megasperma	0.03	0.0–93.3	0.2	0.01–9.4	0.03	0.0–55.8
P. plurivora	1.1	0.2–5.9	1.4	0.3–6.1	0.6	0.08–3.8
P. primulae	–	–	1.1	0.2–5.9	–	–
P. pseudosyringae	1.02	0.2–5.8	0.8	0.1–5.8	1.8	0.6–5.2
P. psychrophila	1.2	0.2–6.0	0.4	0.02–6.8	0.01	0.0–97.8
P. quercina	1.0	0.2–5.9	0.05	0.0–64.3	0.4	0.03–4.0
P. trifolii	–	–	–	–	0.08	0.001–11.1
**New**						
P. andina	–	–	–	–	0.02	0.0–81.0
P. taxon *castitis*	–	–	0.003	0.0–100.0	0.04	0.0–41.3
P. citricola	1.02	0.2–5.8	0.6	0.1–5.9	0.6	0.08–3.8
P. fragariaefolia	–	–	–	–	0.03	0.0–71.6
P. gregata	0.02	0.0–99.1	–	–	–	–
P. obscura	0.007	0.0–99.9	0.2	0.006–8.8	0.05	0.0–29.1
P. 'ohioensis'	1.6	0.4–6.4	–	–	0.5	0.06–3.8
P. pinifolia	–	–	–	–	0.02	0.0–72.9
P. pisi	0.01	0.0–99.9			0.02	0.0–82.3
P. pistaciae	0.2	0.002–12.0	0.007	0.0–99.9	0.06	0.0–19.8
P. ramorum	–	–	–	–	1.01	0.2–4.2

P. siskiyouensis	0.009	0.000–99.9	–	–	–	–
P. syringae	6.6	3.3–12.8	2.6	0.9–7.7	1.2	0.3–4.4
***Halophytophthora* and *Nothophytophthora* species**						
H. fluviatilis	6.2	3.1–12.3	0.5	0.04–6.1	1.4	0.4–4.7
N. amphigynosa	–	–	–	–	0.01	0.0–97.1
N. intricata	0.4	0.03–6.3	0.06	0.0–50.6	0.08	0.001–11.5
N. ‘irlandica’	–	–	–	–	3.6	1.8–7.6
N. ‘liri’	–	–	–	–	0.4	0.04–3.8

Table S3. Association between *Phytophthora* species during autumn and environmental factors. Association between the relative abundance of *Phytophthora* species during autumn along the altitudinal gradient and environmental factors other than climate. Estimate and *p* value of each association is shown. Empty cells indicate species that were not detected during autumn along the altitudinal gradient. The “streams” column indicates the number of streams in which a species was detected. The total number of streams was 118. The “climate” column shows the lowest *p* value of the association between the relative abundance and either temperature or precipitation. Values shown in bold indicate significant associations at $p < 0.05$.

Excel file “Supplementary_Tables_S3_S4”

Table S4. Association between *Phytophthora* species during spring and environmental factors. Association of the relative abundance of *Phytophthora* species during spring along the altitudinal gradient with environmental factors other than climate. Estimate and *p* value of each association is shown. Empty cells indicate species that were not detected during spring along the altitudinal gradient. The “streams” column indicates the number of streams in which a species was detected. The total number of streams was 118. The “climate” column shows the lowest *p* value of the association between the relative abundance and either temperature or precipitation. Values shown in bold indicate significant associations at $p < 0.05$.

Excel file “Supplementary_Tables_S3_S4”
